# Evaluation of Sequencing Library Preparation Protocols for Viral Metagenomic Analysis from Pristine Aquifer Groundwaters

**DOI:** 10.3390/v11060484

**Published:** 2019-05-28

**Authors:** René Kallies, Martin Hölzer, Rodolfo Brizola Toscan, Ulisses Nunes da Rocha, John Anders, Manja Marz, Antonis Chatzinotas

**Affiliations:** 1 Helmholtz Centre for Environmental Research - UFZ, Department of Environmental Microbiology, 04318 Leipzig, Germany; rodolfo.toscan@ufz.de (R.B.T.); ulisses.rocha@ufz.de (U.N.d.R.); johnanders@posteo.de (J.A.); antonis.chatzinotas@ufz.de (A.C.); 2 Friedrich Schiller University Jena, RNA Bioinformatics and High-Throughput Analysis, 07743 Jena, Germany; martin.hoelzer@uni-jena.de (M.H.); manja@uni-jena.de (M.M.); 3 European Virus Bioinformatics Center, 07743 Jena, Germany; 4 Bioinformatics Group, Department of Computer Science and Interdisciplinary Center for Bioinformatics, University Leipzig, 04081 Leipzig, Germany; 5 German Centre for Integrative Biodiversity Research (iDiv) Halle-Jena-Leipzig, 04103 Leipzig, Germany

**Keywords:** viral metagenome, groundwater, aquifer, AquaDiva, sequencing library preparation

## Abstract

Viral ecology of terrestrial habitats is yet-to be extensively explored, in particular the terrestrial subsurface. One problem in obtaining viral sequences from groundwater aquifer samples is the relatively low amount of virus particles. As a result, the amount of extracted DNA may not be sufficient for direct sequencing of such samples. Here we compared three DNA amplification methods to enrich viral DNA from three pristine limestone aquifer assemblages of the Hainich Critical Zone Exploratory to evaluate potential bias created by the different amplification methods as determined by viral metagenomics. Linker amplification shotgun libraries resulted in lowest redundancy among the sequencing reads and showed the highest diversity, while multiple displacement amplification produced the highest number of contigs with the longest average contig size, suggesting a combination of these two methods is suitable for the successful enrichment of viral DNA from pristine groundwater samples. In total, we identified 27,173, 5,886 and 32,613 viral contigs from the three samples from which 11.92 to 18.65% could be assigned to taxonomy using blast. Among these, members of the *Caudovirales* order were the most abundant group (52.20 to 69.12%) dominated by *Myoviridae* and *Siphoviridae*. Those, and the high number of unknown viral sequences, substantially expand the known virosphere.

## 1. Introduction

Groundwater systems are important compartments of the global hydrological cycle. They donate about 30% of all freshwater sources [1] and provide important ecosystem services. For example, purification and storage of water, active biodegradation of anthropogenic contaminants and nutrient recycling [2]. Many of these services are directly linked to the presence of microorganisms [2,3]. Studies in particular in marine systems have significantly contributed to a better understanding of viruses and their impacts on the mortality, diversity and genetic landscape of their microbial hosts [4,5,6]. However, only recently, and only in a limited number of surveys, has the potential role of viruses been explored in terrestrial subsurface systems [7,8,9,10,11].

In theory, metagenomics enables the identification and genomic characterisation of all (micro)organisms present in a sample, including viruses [12]. However, the proportion of viral sequences within a metagenome is usually far lower than for other organisms, leading to limitations in their detection. Especially, pristine aquifers are characterised by low microbial biomass and low abundances of virus particles [9,13,14], which might make their detection even more difficult. Size filtration or density-based enrichment methods are therefore widely used to concentrate virus particles from environmental samples [15,16]. However, a significant obstacle in applying metagenomics for pristine aquifers is the still too low amount of DNA required for the direct sequencing of such samples, making amplification techniques mandatory to further enrich viral nucleic acids. It is however widely known that DNA amplification is a source of bias that may lead to inaccurate conclusions after sequence analysis [17]. Three amplification techniques are commonly used to enrich low amounts of DNA [17,18], i.e., (i) linker amplification shotgun libraries (LASL) [19,20]; (ii) sequence-independent, single-primer amplification (SISPA) [21,22]; and (iii) multiple displacement amplification (MDA) [23,24]. Each method has its own potential source of bias. LASL relies on DNA fragmentation and subsequent linker ligation to blunt-end repaired DNA molecules prior to amplification, using primer oligos that bind to the linker sequences [19,25]. Linker ligation efficiency might be one source of bias, especially for very low amounts of DNA [26]. However, previous studies demonstrated that as little as a few pg to ng of DNA is sufficient for low amplification biases [26,27]. LASL may, in addition, be inefficient in recovering ssDNA viruses due to the double-stranded nature of linker DNA molecules [28] though this has recently been overcome with an adapted LASL protocol [29]. SISPA is built upon the use of pseudo-degenerated primer oligonucleotides, containing a stretch of random nucleotides at their 3’-end and a defined sequence at their 5’-end [21], and has successfully been applied to recover both RNA and DNA virus sequences [22,30]. It has, however, been reported that SISPA has a strong amplification bias resulting in an uneven sequencing read distribution and hence overrepresentation of some genome parts while other parts were completely uncovered. In addition, SISPA negatively affects the detection of low abundant genomes [31]. MDA works under isothermal conditions [32] with very low amounts of input DNA, random hexamer primer oligonucleotides and high fidelity as well as strand displacement functions of the phi29 polymerase [23]. Several sources of bias have been identified for phi29 amplification, including chimera formation [33], discontinuous amplification of linear DNA molecules [34] and preferential amplification of circular ssDNA molecules [35]. Recent studies evaluated different library preparation protocols using low input-DNA to assess the reconstruction of microbial communities from metagenomes [36,37]. Similar studies have been performed for the identification of virus sequences from, for example, seawater and human samples [17,35]. Despite these advances, to our knowledge no study has to date assessed and benchmarked sequence library preparation protocols for the identification of viral sequences from pristine aquifer groundwaters.

The Hainich Critical Zone Exploratory (Hainich CZE) in central Germany is an infrastructure designed to, among others, investigate the diversity, identity and abundance of microorganisms in the Hainich aquifers. In addition, analysis of metabolic potential and activities of microorganisms will be linked to physico-chemical parameters in spatial and temporal scales [38]. Here we sampled three carbonate-rock aquifer assemblages of the Hainich CZE, which represent a pristine and uncontaminated aquifer [38]. One problem in obtaining viral sequences from groundwater samples is the low amount of DNA (usually a pico- to few nanograms) that was extracted from isolated virus particles. The aim of this project is therefore two-fold. The first aim is to evaluate different DNA amplification techniques that may offer a sufficient amount of DNA for high-throughput sequencing. In addition, these methods should have a low amplification bias to reflect the natural diversity of the analysed samples. The second aim consisted of evaluating different viral sequence recovery tools to provide first insights on which viruses are present in the Hainich CZE groundwater aquifers.

## 2. Materials and Methods

### 2.1. Sample Collection

Groundwater samples were taken from three Hainich CZE aquifer wells in Thuringia, Germany, within the framework of the Collaborative Research Centre AquaDiva (http://www.aquadiva.uni-jena.de) (CRC 1076) [38]. The sampling site was located in the agriculturally used midslope and footslope regions of the Hainich low-mountain range. The three wells were drilled to depths of 50 m (H53), 65 m (H52) and 88 m (H51). H53 and H52 reflect anoxic conditions while oxic conditions prevailed for H51. A detailed description of hydrochemical and geostructural parameters can be found elsewhere [39,40].

Ten liters of groundwater (with approximately 2.3 × 10^5^ (SD: 1.2 × 10^4^) viral particles per milliliter) were collected from each well during a sampling campaign in May 2015. Water was filtered through 200 nm pore filters using a cross-flow system (Sartorius, Göttingen, Germany). Samples were then enriched for viral particles by filtration through 35 kDa filters using the same system. Approximately 60 mL were retained and further concentrated by ultracentrifugation at 22,000× *g* for 2 h and 4 °C. The viral particle containing pellet was resuspended in 500 µL TM buffer (50 mM Tris HCl, 10 mM Magnesium sulfate at pH 7.5). One volume of chloroform was given to the samples to remove microsized prokaryotes. The upper phase, intended for DNA extraction, was treated with DNase I to remove free DNA.

### 2.2. DNA Extraction, Library Construction and Sequencing

Viral DNA was extracted as described previously [20]. Viral DNA concentration was determined using the Qubit^®^ dsDNA HS Assay Kit (Thermo Fisher Scientific, Waltham, MA, USA) resulting in total DNA amounts of 31.8 ng (H51), 5.4 ng (H52) and 25.9 ng (H53). DNA was divided into four parts to prepare four libraries for each sample. Non-amplified shotgun libraries (NASL): using a Covaris ultrasonicator, DNA was sheared to approx. 350 bp fragments and libraries were prepared with a TruSeq DNA PCR-Free Library Prep Kit (Illumina, San Diego, CA, USA) according to the manufacturer’s instructions. Linker amplification shotgun libraries (LASL): DNA was sheared to approx. 350 bp fragments as mentioned above and LASL was performed with a NEBNext Ultra DNA Lib Prep Kit (New England Biolabs, Ipswich, MA, USA) as recommended by the manufacturer including 12 PCR cycles to enrich adaptor-ligated DNA. Single-primer amplification (SISPA): PCR was performed by ten cycles using random octamer primers that were linked to a specific primer sequence followed by amplification using a 1:9 mixture of random octamers and a primer targeting the specific primer sequence as described previously [41]. Multiple displacement amplification (MDA): DNA was subjected to phi29 amplification at 25 °C for 8 h using the illustra GenomiPhi V2 DNA Amplification Kit (Thermo Fisher Scientific) as described in the manual. PCR amplicons for the latter two libraries were purified using the Sureclean reagent (Bioline, Luckenwalde, Germany), fragmented as described above and libraries were prepared as described for NASL. Sequencing was performed on one lane of an Illumina HiSeq 2500 system to generate 100-bp paired-end reads.

### 2.3. Sequencing Read Processing and Assembly

PhiX contaminants were removed, SISPA primer sequences were clipped and raw sequencing reads were quality checked using Trimmomatic [42] and low-quality bases were trimmed from both ends. Reads were screened with a 4-base wide sliding window until the remaining sequences had a Phred-score of at least 15 and a minimum length of 36 nt. Sequencing read redundancy was identified by clustering at 90% sequencing read identity using CD-hit v.4.6 [43,44].

Sequencing reads were independently assembled for each sampling site and library preparation using metaSPAdes [45,46] and SOAPdenovo-Trans [47]. In addition, cross-assemblies were performed for each sampling site including all reads from LASL, SISPA and MDA libraries. We used the transcriptome assembler SOAPdenovo-Trans in addition to SPAdes because recent analyses revealed this assembly tool as very efficient for the assembly of RNA virus genomes [48]. Further analyses suggested this might be also true for the assembly of DNA virus genomes.

### 2.4. Viral Contig Recovery

Three viral sequence identification tools were used to recover viral contigs, i.e., VirSorter [49], VirFinder [50] and VrAP (https://www.rna.uni-jena.de/research/software/vrap-viral-assembly-pipeline/). VirSorter is based on the identification of viral hallmark genes, enrichment in hypothetical proteins and other viral signatures [49]. Only contigs identified as VirSorter categories 1 and 2 (higher confidence predictions) were retained for further analysis. VirFinder is a kmer based tool for the identification of viral contigs from metagenomes with improvements especially for the detection of short viral contigs [50]. Contigs with a *p*-value < 0.01 were used for further analysis. These two detection tools were completed by using VrAP, a novel de novo genome assembly pipeline especially designed for viruses. The pipeline is able to assemble complete genomes of viruses representing new strains and species, as well as prototypes of new genera and families. VrAP is based on the genome assembler SPAdes [45] combined with an additional read correction [51,52] and several filter steps. The pipeline classifies the contigs to distinguish host from viral sequences by annotation and open reading frame (ORF) density scores. By applying the ORF density method we were able to identify potential novel viruses without any sequence homology to known references (manuscript in preparation).

### 2.5. Virome Diversity Measures and Comparison of Library Preparation Methods

Nonpareil [53,54,55] was used with default settings to estimate diversity and coverage of virome datasets. Viral reads present in one or more datasets reflecting LASL, SISPA and MDA per sampling site were identified as follows. Redundancy was removed for each dataset by CD-hit-est clustering at 95% identity. A database was created containing all viral contigs and, using Bowtie2 [56], read cluster per library preparation method and sampling site were mapped to the database. Mapped clusters were extracted, counted and overlapping information were generated using SAMtools [57]. Viral contigs were compared between sites by an all-versus-all clustering approach (95% identity) with CD-hit-est-2D [44].

Venn diagrams were computed in R [58] using the package “venneuler” (https://cran.r-project.org/web/packages/venneuler/index.html).

### 2.6. Viral Taxonomic Assignment

All viral contigs per sampling site, i.e., contigs identified from all virus identification tools and library preparation methods, were combined (resulting in three datasets) and redundancies were removed by clustering with CD-hit-est at 95% nt identity. Open reading frames (ORFs) were translated from these contigs using prodigal [59] and aligned to a viral RefSeq protein database (February 2019) using DELTA-BLAST [60] with an e-value cut off of 10^−3^. Hits were sorted by *e*-value and bit score and ORFs with most significant hits were aligned to the respective contigs using an in-house python script (Supplementary Information), resulting in one hit per contig. Gene sharing networks based on shared protein clusters (PCs) between viral genomes were calculated with vConTACT2 [61,62] on the iVirus platform [63] and were displayed with Cytoscape [64]. DNA contamination from cellular organisms was determined using EMIRGE [65].

### 2.7. Data Availability

Sequence read raw data have been made available at Sequence Read Archive accession: PRJNA530103.

## 3. Results

### 3.1. Raw Sequencing Output Statistics

The first aim of this study was to evaluate different DNA amplification techniques that may result in a sufficient amount of viral DNA for high throughput sequencing. We therefore compared three DNA amplification methods, i.e., LASL, SISPA and MDA. NASL was used as control.

MDA produced highest (quality trimmed) sequencing read numbers followed by SISPA and LASL as compared to NASL that exhibited lowest read numbers (Table S1). Significant differences (ANOVA) in quality-trimmed sequencing output were observed between NASL-MDA, NASL-SISPA and SISPA-MDA (Table 1).

Read quality of all libraries was >97% except for libraries H51 LASL and H52 LASL for which 33.78% and 28.13% of the reads were discarded after quality trimming. However, no significant differences (ANOVA) in quality between any of the library preparation methods was observed.

PCR amplification bias may influence the evenness among sequencing reads. For example, GC-rich primers and primers with GC-stretches at their 3’-end, both present in a random primer mix, may anneal more efficiently to a target sequence than AT-rich primer oligos do. As a result, amplicons amplified from such target sequences may be favored during the amplification process what in turn leads to high numbers of identical or related DNA molecules. We therefore clustered all quality-trimmed sequencing reads with a 90% cut-off to remove this redundancy. LASL libraries produced the lowest redundancy (41.7 to 60.7% relative proportion of clusters to sequencing read numbers), with significant differences not only to non-amplified libraries (10.8 to 21.3 relative proportion of clusters to sequencing read numbers) but MDA libraries (9.1 to 17.0% relative proportion of clusters to sequencing read numbers) and SISPA libraries (5.9 to 7.4 relative proportion of clusters to sequencing read numbers) (Table 1). These data suggest an amplification bias during PCR with random primer oligomers. However, MDA libraries (together with LASL libraries) still resulted highest average numbers of read clusters (Table S1). The presence of many repetitive and homopolymeric sequencing reads (possibly sequencing artefacts) may explain the low proportion of clustered reads in NASL libraries.

### 3.2. Data Set Comparison

**Figure 1:**
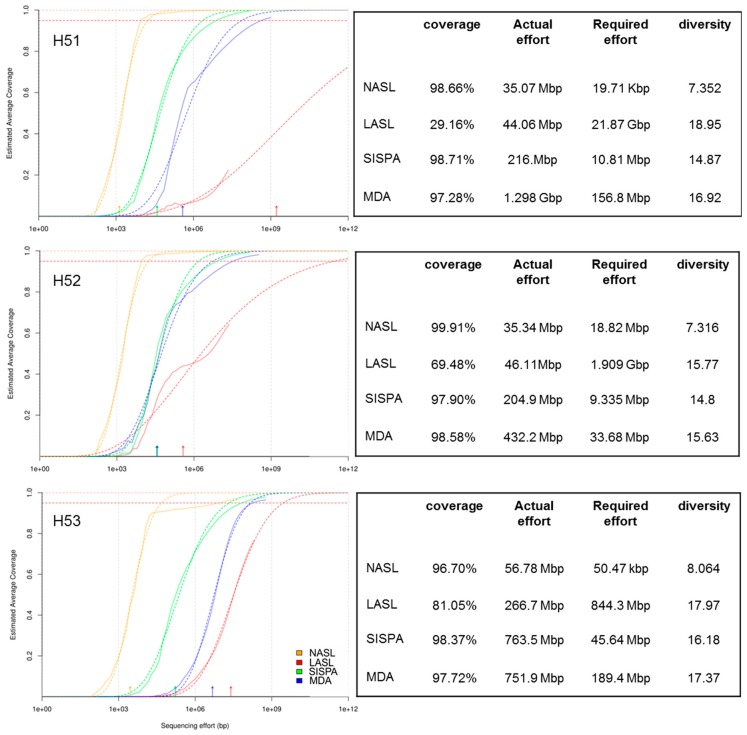
Comparison of Hainich groundwater viromes diversity and coverage as function of sequencing effort using Nonpareil curves [53,54,55]. Estimated coverage is shown as dotted lines, true coverage as solid lines. Estimated diversity is shown with arrows on the *x*-axis. Horizontal dotted line shows 95% coverage. Viral metagenome coverage, actual sequencing effort, required sequencing effort and kmer-based diversity for each library are shown in the right panel.

We used Nonpareil, i.e., a kmer based approach that examines the degree of overlap among individual sequence reads, to determine redundancy [53,54,55] among the individual reads to further assess the average coverage created from the different library data sets. NASL, SISPA and MDA libraries seem to reach a nearly full coverage while LASL libraries vary between ~20 to 80% coverage (Figure 1). However, diversity among libraries increased from NASL to SISPA, MDA and LASL, the latter being the most diverse libraries (Figure 1). These results strongly indicate the target discrimination of SISPA and MDA during PCR that results in uneven coverage of the viral metagenomes and in addition, may fail to target low abundant sequences. NASL sequencing reads dominantly consisted of repetitive and homopolymeric sequences (see also below), with most likely too low an input of DNA explaining the observed Nonpareil curve for these libraries.

**Figure 2:**
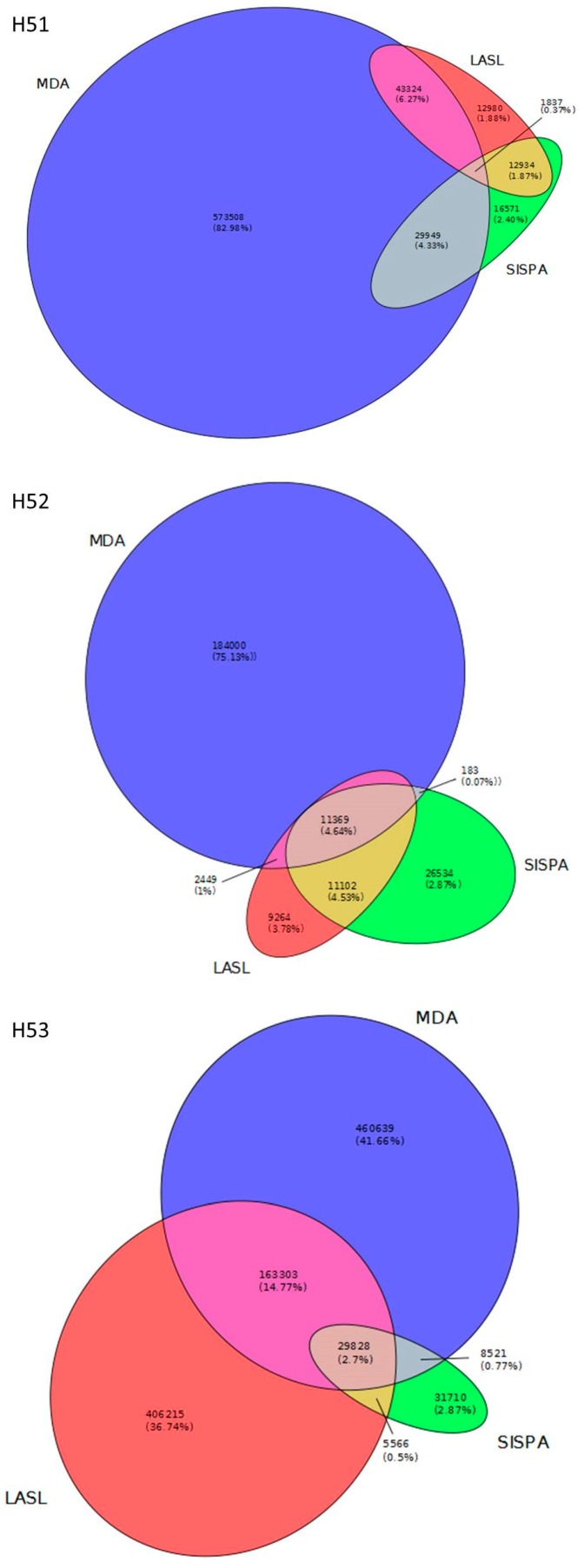
Overlap of sequencing read cluster (90% identity) information identified by library preparation methods, independently shown for each sampling site. Non amplified sequencing libraries (NASL) were not included in the analysis due to the homopolymeric and repetitive nature of sequences obtained from these libraries.

We were further interested in both, the number of viral reads that were exclusively detected by one of the library preparation methods and those reads that were identified from more than one library preparation method. For this, redundancy removed reads (i.e., reads that clustered at 90% identity) of LASL, SISPA and MDA libraries were independently mapped to viral contigs per individual sampling site (i.e., all viral contigs that were identified by the three virus identification tools and cross-assemblies) and counted. MDA libraries produced most reads (average: 350 k) followed by LASL (average: 143 k). Least reads were identified from SISPA libraries (average: 64 k). Overlapping information (reads found in more than one library) was rather low with 0.27 to 4.64% of reads present in all three libraries while 0.07 to 14.77% of reads were identified by two libraries (Figure 2). These data indicate target sequence discrimination between each of the library preparation methods.

### 3.3. Assembly Statistics and Evaluation of Viral Identification Tools

**Figure 3:**
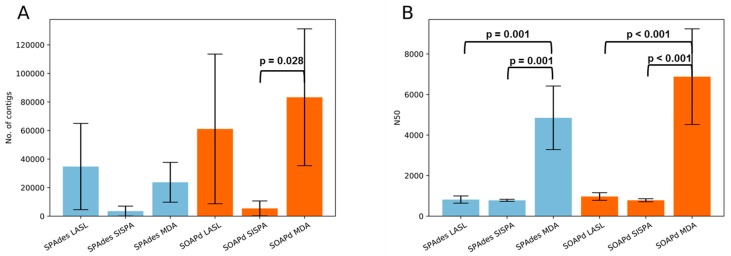
Number of contigs (**A**) and N50 (**B**) produced by sequence library preparation methods and assembly tools. Differences between library preparation methods were tested using analysis of variance (ANOVA). SOAPd: SOAPdenovo-Trans, NASL: non-amplified shotgun library, LASL: linker amplification shotgun libraries, SISPA: single-primer amplification, MDA: multiple displacement amplification.

Using both, SPAdes and SOAPdenovo-Trans, assemblies from non-amplified libraries completely failed due to repetitive and homopolymeric sequences. We therefore excluded these datasets from further analysis. Contig numbers tend to be higher for LASL and MDA libraries than for SISPA libraries (LASL-SISPA: *p* = 0.062, SISPA-MDA: *p* = 0.087; statistical test: one-way ANOVA) when assembled with SPAdes. Similar results were observed for SOAPdenovo-Trans assemblies (LASL-SISPA: *p* = 0.052, SISPA-MDA: *p* = 0.028; statistical test: one-way ANOVA). In addition, MDA library assemblies produced longer contigs (N50) when compared to LASL and SISPA (*p* = 0.001 (SPAdes), *p* < 0.001 (SOAPdenovo-Trans); statistical test: one-way ANOVA) (Figure 3, Table S2). A comparison (student’s t-test) of the two assembly tools showed that SOAPdenovo-Trans may tend to produce longer contigs (*p* = 0.084), while there is no significant difference in the average contig size (N50) (*p* = 0.2972).

**Figure 4:**
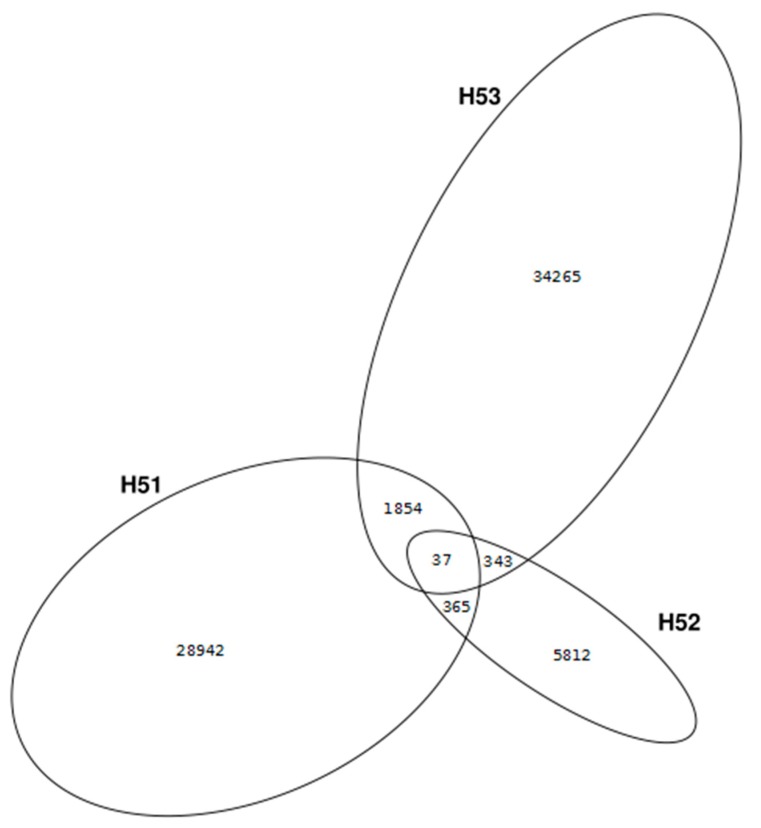
The venn diagram presents numbers of unique and shared viral contigs among the different viromes. Cross-assembled viral contigs (as identified by VirSorter, VirFinder and VrAP) were compared between sites by an all-versus-all clustering approach (95% identity) with CD-hit-est-2D [44].

Viral contigs (as identified by VirSorter, VirFinder and VrAP obtained from cross-assemblies) were clustered at 95% identity to determine a core set of sequences among the sampling sites. Only 37 contigs (0.5%) were shared by the three viromes indicating there is at least a minor common core set in the groundwater aquifers. The amount of shared contigs increased from 0.85% (H51 and H52) and 1.04% (H52 and H53) to 2.85% (H51 and H53) when two viromes were compared. However, the majority of viral contigs is exclusive for the respective virome (Figure 4). The overall viral contig number from H52 is rather low compared to H51 and H53, most likely due to the lower amount of DNA extracted from this sample. This might explain the lower contig overlap of H52 with H51 and H53, respectively, than the overlap of H51 and H53.

We used three different viral sequence identification tools that are based on the detection of viral hallmark genes (VirSorter), kmer distribution (VirFinder) and orf density (VrAP) (see more detailed description in the Materials and Methods section). VirFinder and VrAP significantly identified a higher number of viral contigs than VirSorter (One-way ANOVA *p* < 0.001). The size of viral contigs obtained by VirSorter and VirFinder were in contrast significantly higher than for VrAP (one-way ANOVA *p* < 0.05). However, each tool identified viral contigs that were not recognized by the other two revealing an advantage in the use of several identification tools for the recovery of viral sequences.

### 3.4. First Insights into Viral Taxonomic Composition of Hainich Groundwater

**Figure 5:**
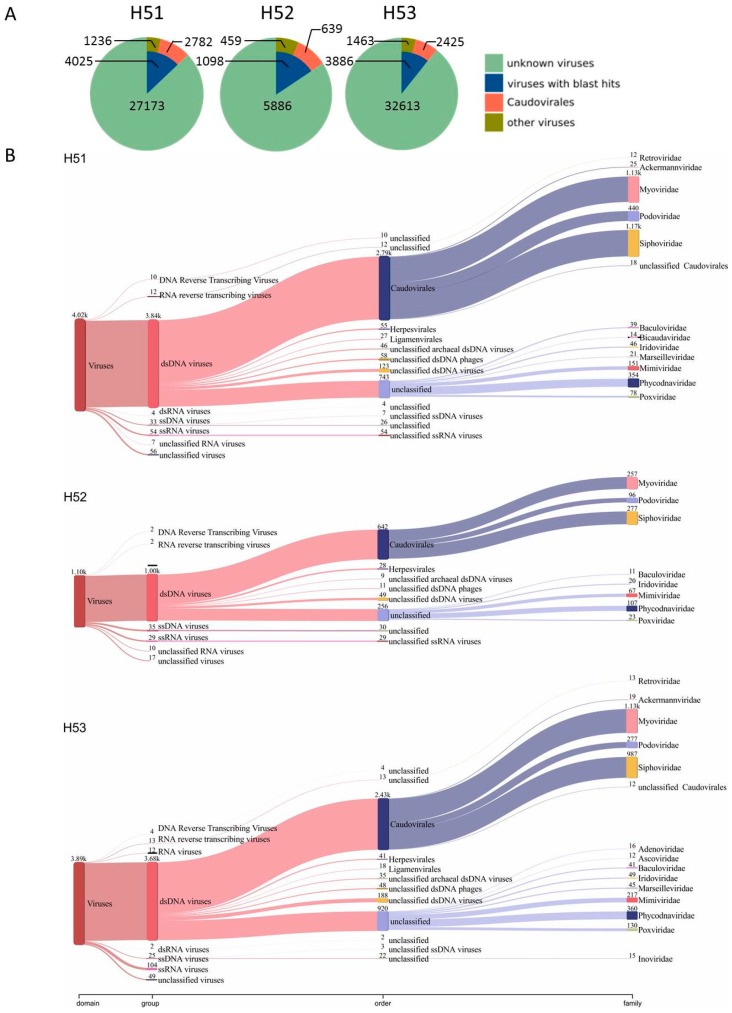
Taxonomic assignment of viral contigs identified from cross-assemblies. (**A**) pie charts present relative and absolute abundance of viral contigs after blastp analysis. (**B**) Taxonomic profile of viral contigs as classified by blastp (viral contigs with blast hits in figure **A**). Data were visualized with Pavian [71].

Using cross-assembled contigs (assemblies including sequencing reads from LASL, SISPA and MDA per sampling site) and a set of three viral sequence recovery tools, we identified 27,173 (H51), 5,886 (H52) and 32,613 (H53) viral contigs from the Hainich groundwater samples (Figure 3; Table S3). These contigs were assembled from 31.19% (H51), 52.08% (H52) and 28.41% (H53) of the quality trimmed sequence reads. Among these reads we identified 19 Small subunit ribosomal ribonucleic acid sequences (8 bacterial 16S, 11 unclassified) demonstrating a low contamination with DNA from cellular organisms. Only 14.81% (H51), 18,65% (H52) and 11.92% (H53) of the viral populations could be assigned to taxonomy using delta-blast (Figure 5A). Most of them were assigned to dsDNA viruses dominated by the order *Caudovirales* (H51: 69.12%, H52: 58.20%, H53: 62.40%). Within the *Caudovirales,* members of the *Myoviridae* (40 to 46.5%) and *Siphoviridae* (40.7 to 41.9%) families were most abundant (Figure 5B, Figure S1). These findings are not surprising since *Caudovirales* have previously been presented as the most abundant group of viruses in environmental ecosystems [8,66,67]. Other identified dsDNA virus sequences belonged, for example, to the amoeba infecting giant virus families *Marseilleviridae* and *Mimiviridae*, to the algae infecting *Phycodnaviridae* family whose hosts has been shown to be present in groundwater [68], and invertebrate-infecting viruses such as *Iridoviridae* and *Poxviridae* (Figure 5B). Surprisingly, we identified only a small number of circular ssDNA viruses (Figure 5B). These viruses have been revealed as an abundant group in other environments [69,70]. We used Phi29 polymerase in MDA that preferentially amplifies circular ssDNA [35] and one could expect a bias towards overrepresentation of circular ssDNA genomes. Although this study is only a first snapshot into the Hainich groundwater virome we speculate that circular ssDNA viruses are rare in this environment. A small fraction of these DNA viromes was assigned to RNA viruses, most likely due to PCR errors and incomplete/erroneous virus reference databases.

**Figure 6:**
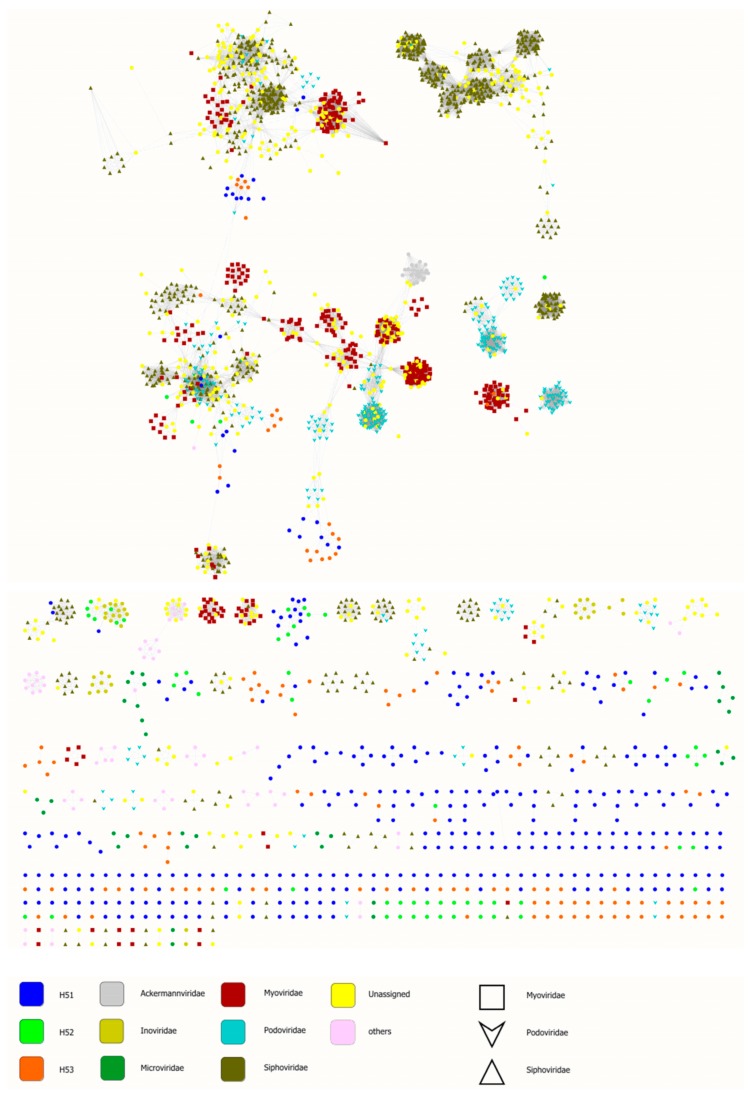
A network analysis of shared predicted protein content between viral RefSeq database and Hainich viral populations. Nodes (circles) indicate contigs and shared edges (lines) indicate shared protein content. Data were analysed using vConTACT2 [61,62] and displayed with cytoscape [64].

However, a high number of blast-based taxonomy assigned contigs could not be affiliated to deeper taxonomic levels but have similarity to unclassified viruses present in the viral RefSeq database (Figure 5B). These findings, together with the huge number of unknown viral contigs (without any blast hit) reveal substantial genomic and taxonomic diversity in Hainich groundwater viromes, as observed also in other environments [66,72]. To further investigate the similarity of Hainich groundwater viromes to viral RefSeq database, we used a genome-based network analysis of their shared protein content (Figure 6) [61,62]. This analysis groups viral contigs at the approximately genus level into viral clusters [61,62,73]. In total, 539 viral clusters were identified. Of those, Hainich viral contigs were found in 191 clusters, 183 of them were exclusive to Hainich viromes, among those 95 clusters exclusive to H51, 8 clusters to H52 and 23 clusters to H53. In addition, approximately 34% (H51), 64% (H52) and 63% (H53) of viral protein clusters were present in at least one other Hainich groundwater sample, suggesting some sequence conservation across these samples.

## 4. Discussion

Viruses play a key role in ecosystems, with most of them infecting microbes. They directly affect their hosts by lysis and horizontal gene transfer, and hence are responsible for changes in microbial community structure and composition what in turn has consequences on biogeochemical cycles and food web structures [4,5,6,74]. Viral metagenomics has been increasingly used to unravel viral community composition and interactions with their hosts from different ecosystems, such as marine environments and soil [66,67,72]. The terrestrial subsurface including groundwater ecosystems is at present yet underexplored [7,8,10,11,75]. A common problem is the relatively low biomass present in these difficult to obtain samples, which in return, results in only low amounts of DNA not sufficient for standard preparation of metagenome sequencing libraries [9,13,14]. Efforts have been undertaken to overcome this problem, including DNA enrichment using different DNA amplification techniques [17,18,19,22]. Each of these methods has its own advantages and limitations making it difficult to provide a standard protocol. Benchmark tests should therefore be performed when investigating new sample types.

Sampling procedure, virus particle isolation and nucleic acid extraction protocols are potential sources of bias [17] that have to be considered prior to sampling. Here, we focused on non-enveloped DNA viruses that passed a pore size of 200 nm after filtration and performed a benchmark study to find a method of choice to enrich viral DNA that is sufficient for sequencing. We furthermore intended to get a first snapshot of the viruses present in Hainich groundwater aquifers.

We used three DNA amplification methods, i.e., LASL, SISPA and MDA to compare one another and with NASL, using three groundwater samples. Although NASL resulted in some sequencing output none of the reads could be used for further analysis (assembly, virus sequence identification) due to their repetitive and homopolymeric nature; demonstrating that direct sequencing of NASL is not feasible with low DNA amounts. According to the Nonpareil curves, LASL was the method with the lowest amplification bias since the curves were located rightward in the plots indicating a higher diversity than for SISPA and MDA (Figure 1). Nonpareil curves for SISPA and MDA simulate a nearly full sequence coverage that emerge from redundant sequence information (Table 1, Table S1). False sequence coverage interpretation could be a result when data analysis exclusively rely on these library preparation methods. In addition, LASL resulted in the highest number of unique sequencing reads as compared to SISPA and MDA. MDA on the other hand outperformed LASL and SISPA in terms of viral contig numbers and their average contig size. In addition, MDA performed (at least in two samples) much better for taxonomic assignments in the case of *Caudovirales* families, which were dominant among the viral contigs with taxonomic affiliation (Figure S1). Considering the amount of unique viral reads per method and their low overlap (Figure 2), together with the results from cross-assemblies, it became apparent that none of the here tested DNA enrichments methods could completely detect viral sequences from pristine groundwater. However, SISPA even underperformed in terms of sequencing output, diversity and assembly statistics. Metagenomic benchmark studies using both, microbial mock communities and marine samples demonstrated the use of Mondrian and Illumina Nextera XT technologies produced high quality metagenomes from even femtogram-input DNA libraries [36,37]. These library preparation methods are comparable with the LASL protocol used in this study because all these methods use linker ligation on fragmented or tagmented DNA prior to amplification for generation of sequencing libraries. The low bias introduced by LASL on virus enriched groundwater samples from our work is consistent with these previous studies on prokaryotic metagenomes. In addition, other studies on viromes from marine and human samples showed substantial differences with respect to diversity, assembly output, types and ratio of viral sequences between LASL and MDA [18] and an outperformance of MDA over SISPA [17]. However, these studies observed an overrepresentation of circular sequences in MDA libraries as compared to LASL and SISPA. In contrast, our data identified only a few contigs that belong to circular ssDNA viruses (see also discussion below). We therefore suggest the combined use of LASL and MDA for future analysis of viral communities from pristine groundwater aquifers.

SOAPdenovo-Trans produced more contigs than SPAdes. However, average contig size was similar (Figure 3, Table S2). A combination of the assembly output seems to produce most comprehensive results but might also introduce unnecessary redundancy. Assembly for metagenomic data is already difficult, but appear to be more complex for viruses with their possibly more uneven genome coverage. Specialized tools are needed for the (de novo) assembly of viral sequences from metagenomic data [76]. The lower number of contigs for H52 could be a result of the lower amount of DNA extracted from this sample as compared to H51 and H53. Future studies will reveal whether there is a correlation between input DNA amount and contig numbers, including replicates and different yields of DNA input.

There is a high number of virus identification tools available, with all of them having their limitations [77]. We decided to use VirSorter [49], VirFinder [50] and VrAP. The latter two do not rely on database matches, increasing the chance to detect novel viruses not related to those present in public databases. Using our dataset, each tool exclusively identified some viral contigs demonstrating a combination of different virus identification tools increases the number of recovered viral contigs as also suggested previously [59,78]. However, the number of viral contigs was lower than the total number of contigs (compare Table S2 and Table S3). The experimental procedure included several steps to enrich virus particles, i.e., size filtration, chloroform treatment to remove most small-sized bacteria and digestion of free DNA that is not protected by a protein shell. Although some non-viral sequences might still be present after such methodology, one could assume the majority of the dataset consists of viral sequences and consequently includes a high number of viral contigs not recovered by one of the detection tools. Efforts should be undertaken, e.g., using machine learning, to overcome these likely limitations [78,79].

Like in many environmental studies, the taxonomy of most viral contigs remained unknown as demonstrated by blast and network analysis (Figure 5, Figure 6) [8,10,66]. Members of the order *Caudovirales* were dominating among viral contigs with taxonomic assignment. This group of tailed viruses infects a wide variety of bacteria and has been shown as one major group present in environmental ecosystems [8,66,72,80,81]. Another group of commonly highly abundant viruses, i.e., circular ssDNA viruses of the families *Microviridae* and *Circoviridae* [69,70,82], were almost entirely absent in our dataset. This is in contrast to previous results from groundwater aquifers where these viruses even dominated over dsDNA viruses among the classified sequences [10]. A technical bias seems to be unlikely since MDA is known for preferential amplification of these target sequences [35]. Future analyses including spatial and temporal variation will reveal whether these viruses are rare in pristine groundwater. We further identified viruses infecting algae, invertebrates and microeukaryotes, among the latter, contigs similar to giant viruses from the *Mimiviridae* family. These viruses should, by default, not be detected after 200 nm pore size filtration. A possible explanation could be sequence similarity of conserved mimivirus orfs, such as polymerases, to yet unknown viruses [83,84].

We show viral metagenome libraries can be produced from pristine aquifer groundwaters and suggest a combination of LASL and MDA to enrich viral DNA from these samples and to diminish an amplification bias that may occur during enrichment. We further identified new viral sequences that will help to understand the role of viruses in pristine groundwaters.

## Figures and Tables

**Table 1:** *P*-values of analysis of variance (ANOVA) of raw sequencing read and read cluster numbers between the different library preparation methods.

	**Number of Raw Reads**							
Library Preparation	NASL		LASL		SISPA		MDA	
NASL	n/a		>0.05		0.008		0.002	
LASL			n/a		>0.05		0.023	
SISPA					n/a		>0.05	
MDA							n/a	
	**Clusters at 90% Read Identity**							
	Relative proportion				Number of clusters			
Library Preparation	NASL	LASL	SISPA	MDA	NASL	LASL	SISPA	MDA
NASL	n/a	<0.001	>0.05	>0.05	n/a	0.018	>0.05	0.008
LASL		n/a	<0.001	<0.001		n/a	>0.05	>0.05
SISPA			n/a	>0.05			n/a	>0.05
MDA				n/a				n/a

NASL: non-amplified shotgun library; LASL: linker amplification shotgun libraries; SISPA: single-primer amplification; MDA: multiple displacement amplification.

## Supplementary Materials

The following are available online at https://www.mdpi.com/1999-4915/11/6/484/s1, Figure S1: Bubble plot shows families of the Caudovirales order present in each virome., Table S1: Overview of raw sequencing read pair numbers, sequence quality and sequencing read clusters for each prepared sequencing library., Table S2: Assembly statistics for LASL, SISPA and MDA libraries per sampling site., Table S3: Overview of identified viral contigs as per virus identification tool, assembly software, sequencing library and sampling site., Supplementary information: Python script to assign orfs to contigs.
