# Supplementary material for: Evaluation of Sequencing Library Preparation Protocols for Viral Metagenomic Analysis from Pristine Aquifer Groundwaters
Source: Viruses. 2019 May 28;11(6):484. doi: 10.3390/v11060484 (PMC6631259; doi:10.3390/v11060484)
Supplement: Supplementary file 1 [file viruses-11-00484-s001.pdf]

Table S1. Overview of raw sequencing read pair numbers, sequence quality and sequencing read clusters for each prepared sequencing library.

	H51 NASL	H51 LASL	H51 SISPA	H51 MDA
Raw read pairs	347,238	707,163	2,556,023	12,932,443
Quality[1]	99.43%	66.22%	97.40%	99.79%
Read cluster[2]	74,717 (10.8%)	858,145 (60.7%)	300,934 (5.9%)	2,810,818 (10.9%)
	H52 NASL	H52 LASL	H52 SISPA	H52 MDA
Raw read pairs	349,946	859,130	2,411,456	4,290,842
Quality[1]	99.10%	71.87%	97.09%	99.74%
Read cluster[2]	84,425 (12.1%)	716,741 (41.7%)	308,501 (6.4%)	776,688 (9.1%)
	H53 NASL	H53 LASL	H53 SISPA	H53 MDA
Raw read pairs	562,129	2,726,966	8,673,929	7,449,774
Quality[1]	99.23%	99.36%	98.40%	99.83%
Read cluster[2]	239,431 (21.3%)	2,834,872 (52.0%)	1,283,132 (7.4%)	2,539,692 (17.0%)

[1] Percentage of reads retained after quality trimming

[2] Number of unique reads after clustering at 90% sequencing read identity; relative proportion of clusters to sequencing read numbers in parentheses

LASL: linker amplification shotgun libraries

SISPA: single-primer amplification

MDA: multiple displacement amplification

Table S2. Assembly statistics for LASL, SISPA and MDA libraries per sampling site.

	H51 LASL		H51 SISPA		H51 MDA		H51 cross	
	SPAdes	SOAPd	SPAdes	SOAPd	SPAdes	SOAPd	SPAdes	SOAPd
No. contigs	14,268	21,699	1,247	2,156	42,003	142,990	55,172	162,280
>= 1000	50	34	35	35	5,447	4,826	5,575	4,904
>= 5000	4	1	4	6	786	1,064	784	1,087
>= 10k	1	0	3	4	182	330	175	337
N50	627	916	849	890	2,647	4,454	2,414	4,381
	H52 LASL		H52 SISPA		H52 MDA		H52 cross	
	SPAdes	SOAPd	SPAdes	SOAPd	SPAdes	SOAPd	SPAdes	SOAPd
No. contigs	12,517	26,387	960	1,334	8,170	25,627	21,670	51,882
>= 1000	412	233	29	17	863	790	1,324	1,080
>= 5000	3	8	0	0	169	178	174	181
>= 10k	1	2	0	0	94	116	94	116
N50	772	770	762	734	5,737	10,076	1,602	3,193
	H53 LASL		H53 SISPA		H53 MDA		H53 cross	
	SPAdes	SOAPd	SPAdes	SOAPd	SPAdes	SOAPd	SPAdes	SOAPd
No. contigs	77,469	135,200	8,460	12,805	21,008	81,200	80,313	165,733
>= 1000	7,819	6,594	251	214	2,693	2,032	8,608	6,966
>= 5000	226	348	1	2	566	332	692	453
>= 10k	40	81	0	0	240	180	273	201
N50	1,056	1,221	733	727	6,167	6,111	1,364	1,282

LASL: linker amplification shotgun libraries

SISPA: single-primer amplification

MDA: multiple displacement amplification

cross: cross-assembly of all reads obtained from LASL, SISPA and MDA

SOAPd: SOAPdenovo-trans

>=: minimum size of contigs

N50: average contig size

Table S3. Overview of identified viral contigs as per virus identification tool, assembly software, sequencing library and sampling site.

		VirSorter				VirFinder				vrap	
		SPAdes		SOAPdenovo-Trans		SPAdes		SOAPdenovo-Trans			
		No.[1]	N50[2]	No.[1]	N50[2]	No.[1]	N50[2]	No.[1]	N50[2]	No.[1]	N50[2]
H51	LASL	44		0		353		1,784		18	
	SISPA	0		0		188		267		0	
	MDA	43		62		3754		22,847		3,628	
	cross	44	7.9	65	11.2	4255	3.6	24,315	5.9	3,518	1.3
H52	LASL	1		1		200		1,752		0	
	SISPA	0		0		142		122		19	
	MDA	14		17		724		3,422		938	
	cross	15	26.4	17	24.3	1094	21.1	5,077	16.2	781	1.1
H53	LASL	13		15		977		5,429		2,632	
	SISPA	1		0		254		823		97	
	MDA	14		14		556		6,164		2,440	
	cross	22	27.6	19	21.4	1441	6.1	27,132	1.7	2,945	0.9

[1] Number of contigs that were identified as viral
[2] average contig size
LASL: linker amplification shotgun libraries
SISPA: single-primer amplification
MDA: multiple displacement amplification
cross: cross-assembly of all reads obtained from LASL, SISPA and MDA

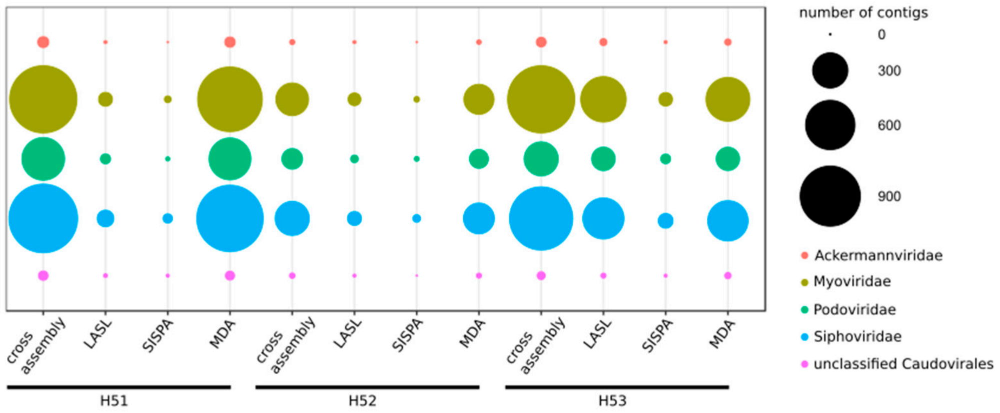

Figure S1. Bubble plot shows families of the *Caudovirales* order present in each virome. Bubble size represents the number of contigs per family. Differences in detection of contigs depending on library preparation are also shown.

Supplementary information

Supplementary data S1. Python script to assign orfs to contigs

```
import os
import sys

# path to the directory where the blast outputs are
blast_outputs="/path/to/directory/Blast_output"

# path to the reference files
# NCBI fullnamelineage.dmp file
full_name="/path/to/directory/fullnamelineage.dmp"

# NCBI prot.accession2taxid file
tax_file="/path/to/directory/prot.accession2taxid"

def grep(query,file):	# this function looks for a query in a file and returns the whole line
		l="a"
		f=open(file,"r")
		while True:			# open and read file until the end
				l=f.readline()		# for each line
				if not l: break
				else:
						if query in l: return l		# if query is in line, return line
		return 0 # if it didnt find anything, return 0

def get_best_blast_hits(blast_file): # this function returns the best blast hits for each contig and
returns the data inside a dictionary
	l="a"
	f=open(blast_file,"r")
	dic={}		# create dictionary (unique keys ;)
	print "fetching best hits for each contig..."
	while True:			# while there is file to read
		l=f.readline()		# read file line by line
		if not l: break		# if line is empty (end of file), break
		else:
			info=l.split("\t")	# if not, split the line in a list using tab as separators
			cntg = info[0].rsplit('_', 1)[0] # removal of the orf number,
			acc_num=info[1].strip()		# the second column is the accession_number
			evalue=float(info[10].strip())		# the 11th column is the evalue
			bitscore=float(info[11].strip())	# the 12t column is the bitscore

		if cntg not in dic:		# for each line, if contig not in dictionary
```

```
                dic[cntg]=[cntg,acc_num,evalue,bitscore]    # store it inside dictioary
            else:
                if evalue < dic[cntg][1]:          # if contig is already there
                    dic[cntg]=[cntg,acc_num,evalue,bitscore]    # if current line has lower eval,
replace
                elif evalue == dic[cntg][1]:             # if evalue is the same
                    if bitscore > dic[cntg][2]:                # compare bitscore. if higher, replace
                        dic[cntg]=[cntg,acc_num,evalue,bitscore]
    return dic              # once the dictionary is done, return as output of function

def import_tax_file(cntg_dic,taxid_file):      # this function mapss the acc_number to a given tax_id
    accnum_cntg_dic={}
    for k,v in cntg_dic.iteritems(): accnum_cntg_dic[v[1]]=k # creates an auxiliary dictionary
    l="a"
    dic={}
        f=open(taxid_file,"r")

    print "mapping acc_num to tax_id..."
        while True:                          # reads taxid file and dumps matching tax_ids inside a dic
                l=f.readline()
                if not l: break
                else:
            info = l.split()
            if info[1] in accnum_cntg_dic:
                dic[accnum_cntg_dic[info[1]].strip()] = info[2].strip()
    return dic
    #print len(dic)
def get_lineage(blast_inp,cntg_tax_dic,tax_name_dic):
    print "dumping to output file..."
    f=open(blast_inp+"_names.tsv","w")

    for k,v in cntg_tax_dic.iteritems():
        #print k,v
        info = k+";"+ tax_name_dic[v]
        f.write(info+"\n")

def get_tax_name_dic(name_file):
    dic = {}
    l="a"
    print "linking tax_id to full name..."
        f=open(name_file,"r")
        while True:
                l=f.readline()
```

```
                if not l: break
                else:
            #print l
            info= l.replace("\n","").replace("|",";").replace("\t","").replace("; ;",";")#.split(";")
            tax = info.split(";")[0]
            #print info
            #info = info.rsplit(';', 1)[0]
            #info.pop(0)
            #print info
            #print ""
            dic[tax]=info[:-1]
    #for k,v in dic.iteritems():
    #       print       k,v
    return dic

blasts=os.listdir(blast_outputs)

# get full names dic
tax_name_dic=get_tax_name_dic(full_name)

for b in blasts:

    blast_inp = blast_outputs+"/"+b
    print "STARTING ",blast_inp

    best_hits_dic=get_best_blast_hits(blast_inp)
    cntg_tax_dic=import_tax_file(best_hits_dic,tax_file)
    get_lineage(blast_inp,cntg_tax_dic,tax_name_dic)

    print blast_inp,"DONE \n--------------------------------"
    #break
```
